# Partnership for Native American Cancer Prevention: Outreach Strategies and Evaluation 2019–2024

**DOI:** 10.1002/cam4.71348

**Published:** 2025-11-12

**Authors:** Nicolette I. Teufel‐Shone, Kelly A. Laurila, Julie S. Armin, Carol Goldtooth, Francine C. Gachupin, Jacquanette R. Slowtalker, Janet R. Yellowhair, Ashley D. Lazaro, Eli BigThumb, Alexis K. Talayumptewa

**Affiliations:** ^1^ Center for Community Health and Engaged Research Northern Arizona University Flagstaff Arizona USA; ^2^ Department of Health Sciences Northern Arizona University Flagstaff Arizona USA; ^3^ Department of Family and Community Medicine, College of Medicine University of Arizona Tucson Arizona USA; ^4^ Arizona, Department of Health Services Phoenix Arizona USA

**Keywords:** cancer, community engagement, education, indigenous, Native American, outreach, strategies

## Abstract

**Aim:**

The Outreach Core of the Partnership for Native American Cancer Prevention (NACP) in Arizona offers an overview of strategies designed to (1) provide cancer‐related education in collaboration with Native Nations and (2) train researchers working with native American communities on tribal oversight of research activities and community‐oriented dissemination.

**Background:**

In the late 1990s, the National Institute of Health began to support an Outreach Core within its Center grants but provided limited guidance on expected activities and goals. NACP has been funded since 2002 and refunded to 2029. The NACP Outreach Core shares strategies implemented from 2019 to 2024. In this 5‐year period, the NACP Outreach Core had four goals with the intention of increasing institutional and community capacity for cancer control activities: (1) Increase researcher, trainee and tribal communities' knowledge of native American cancer‐related issues to enhance institutional and tribal capacity to design sustainable, relevant cancer research and prevention programs. (2) Provide training in research best practices when collaborating with tribal communities. (3) Facilitate community‐based dissemination of NACP research results. (4) Collaborate with native American communities to develop and implement cancer‐related informational activities to benefit native American communities.

**Methods:**

Goal 1 employed 3 strategies: a speakers' series, regular contribution to a week‐long workshop on genomics research in Indigenous communities and development of cancer research resources for Native Nations. Goal 2 involved contributions to a university workshop on native American research protections. Goal 3 was addressed with the development and implementation of community dissemination training for NACP‐funded research teams. Goal 4 focused on building relationships, by responding to Native Nations' requests to present at community conferences and cancer awareness events.

**Results:**

The Indigenous Cancer Prevention Speaker Series was implemented each quarter for 5 years. Through participation in the Summer Internship for INdigenous Peoples in Genomics, NACP provided education on the collection of biospecimens with Indigenous peoples. NACP developed and distributed cancer education resources to tribal health directors across Arizona. NACP designed and delivered virtually, in real time, the Community Dissemination and Application Training to 31 NACP‐funded investigators and provided one‐on‐one consultation to research teams. In collaboration with providers in Native Nations, NACP developed a cancer conversation podcast series, *Taking Care of Us*, reaching national and international listeners. Over 5 years, NACP Outreach participated in 20 community events including cancer town halls, health fairs, radio segments, and cancer‐related fun runs, reaching over 1400 community members.

**Conclusion:**

NACP Outreach Core's range of strategies provided cancer‐related information to institutional scholars and tribal communities and maintained regular, reciprocal communication with Arizona Native Nations. The impact of these activities is difficult to assess and is limited to reach, that is, counts of participants, or “hits” on an online resource. These metrics demonstrate that NACP Outreach products are used but do not track impact. The ability to evaluate impact should be considered when designing outreach strategies. The Outreach Core team's regular connection with native American communities contributes to NACP's long‐term partnership with Arizona Native Nations; community‐university relationships are critical to ensuring receptivity and relevance of outreach activities.

## 1 Introduction

This manuscript describes several signature strategies and programs of the Outreach Core of the National Institute of Health's National Cancer Institute (NIH‐NCI) U54 funded Partnership for Native American Cancer Prevention Partnership (NACP) implemented in the 2019–2024 funding cycle [1]. The rationale, approaches, activities, and outcomes of the NACP Outreach Core are shared to offer ideas to emerging or seasoned health research centers exploring alternate strategies for community engagement. NACP's work has focused predominantly on addressing native American cancer health disparities in Arizona but through partnerships and student engagement has reached other Native Nations throughout the United States (U.S.).

## 2 Overview of the Native American Cancer Prevention Partnership (NACP)

**FIGURE 1:**
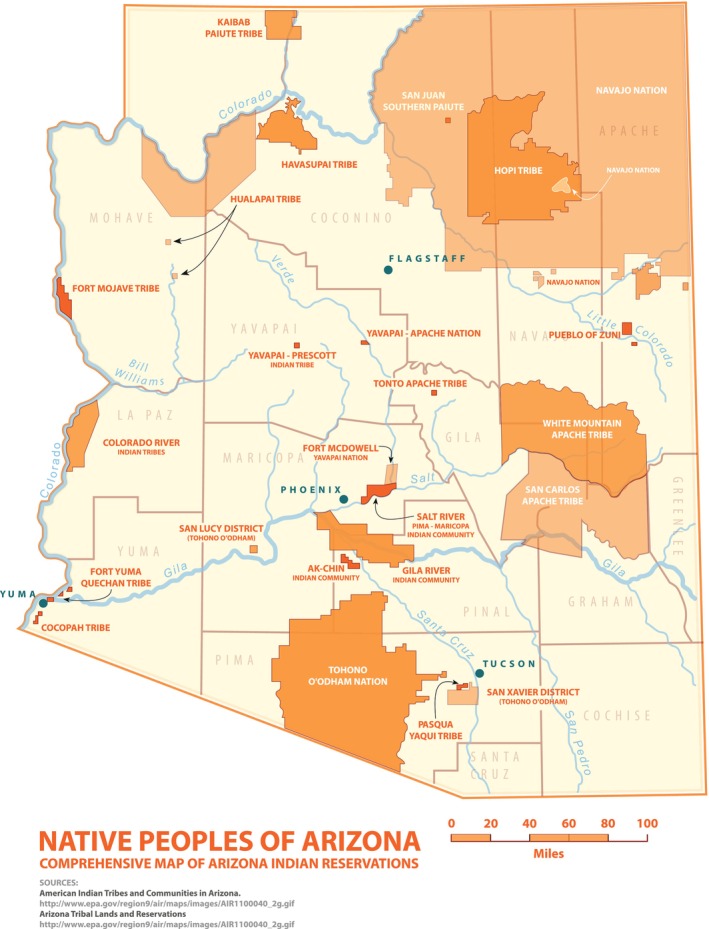
Arizona native nations and counties [3].

The NACP's mission is to alleviate the unequal burden of cancer among Native Americans of the Southwest, with a primary focus on Arizona, through research, training, and community outreach programs in collaboration with the communities served [2]. The NACP began in 2002 to reduce cancer health disparities and is a collaborative partnership between a large NCI‐funded Comprehensive Cancer Center, the University of Arizona Cancer Center (UACC) and a smaller minority‐serving institution, Northern Arizona University (NAU). The institutional match is a requirement of the NCI U54 funding mechanism, Comprehensive Partnerships to Advance Cancer Health Equity (CPACHE) program. Collectively, the NACP has engaged 18 of Arizona's 22 Native Nations through cancer outreach, research, or education (see Figure 1) [4]. NACP has disseminated descriptions and outcomes of these partnerships in national and international Indigenous‐focused scientific and community‐oriented conferences.

NACP is guided by three advisory boards. The Program Steering Committee (PSC) is an external advisory board composed of nationally recognized experts in cancer research, health disparities including native American‐specific cancer disparities, and mentorship of biomedical and social science researchers. The PSC also includes program officials from the NCI's CPACHE program. The Internal Advisory Committee (IAC) members hold key institutional leadership positions within UACC and NAU, and several have supported faculty recruitment efforts important to the goals of NACP. The Community Advisory Board (CAB) is composed of community members that hold leadership positions within AZ Native Nations and are involved in cancer health‐related tribal initiatives.

Beginning in funding cycle 4 (2019), the CAB, which previously only advised the Outreach Core, was elevated to serve at the same level as the PSC and IAC advising Partnership leadership. Specific CAB member responsibilities include: Attend annual CAB meetings with NACP leadership to review progress, priorities, and new directions; Advise, as needed, when issues arise within NACP, including community engagement and consultation with project teams when developing new research initiatives; Attend the annual PSC meeting to provide the PSC with community perspectives on NACP progress; and Review solicited, proposed full and pilot proposals with a focus on responsiveness to community needs and potential for impact on health within Native communities. The CAB has provided a community lens to inform program activities, proposed research projects, identified research priorities, and shared overall community‐based cancer experiences. The NACP Outreach Core members, UACC and NAU faculty and staff, have worked with native American communities on addressing health disparities for several decades and through their networks, nominated many of the CAB members to NACP leadership CAB‐related evaluation strategies and outcomes are not discussed in this paper as they are not part of the NACP Outreach Core, specifically.

## 3 Funding Agency Expectations

In the late 1990s, the National Institute of Health (NIH) began to support an Outreach Core within its Center grants [1]. Until 2024, the descriptions and required activities of this Core have been brief, often 3–4 sentences in comparison to 7–10 sentences for the Administrative and Research/Education Cores. The brevity and broad language may be purposeful, recognizing the diversity of communities and technical assistance needed by the research teams supported by their respective centers. But with the vagueness of the guidelines, the expectations of these emerging outreach cores were not clear. The outcomes of community engagement can be challenging to measure and are often designed to increase familiarity, understand community context and build trust. Outcomes of activities are often process measures of co‐developed interventions including educational materials, designed to support researchers, communities, or both.

An excerpt from the Funding Opportunity Purpose in NCI's 2018 announcement of the CPACHE offers language that suggests the outreach activities should benefit the communities with health disparities [1].

**Outreach Core**: It is expected that both partnering institutions will engage in outreach efforts that would benefit communities with cancer health disparities. Such efforts are strongly encouraged to be connected to cancer‐relevant outreach research (e.g., through pilot projects or research development activities) conducted by the partnership [5].

Yet, the language in the description of the expectations for the outreach research strategy is more expansive and includes benefits to underserved communities, clinicians, and the research enterprise.

**Research Strategy**: Cancer outreach in the context of this FOA is a joint effort between partnering institutions and must include efforts to effectively reach individuals and physicians and should lead to increased use of specific medical procedures that may decrease cancer health disparities. For example, they may lead to identifying, developing, testing, evaluating, and/or refining strategies to disseminate and implement evidence‐based practices (e.g., behavioral interventions; prevention, early detection, diagnostic, treatment and disease management interventions; quality improvement programs) into public health, clinical practice, and community settings. In addition, outreach efforts directed at recruitment and retention of individuals from underserved health disparity populations into prevention, early detection, and therapeutic clinical trials, as well as participation in biospecimen donation research, are strongly encouraged. The proposed outreach efforts are strongly encouraged to be connected to cancer‐relevant outreach research conducted by the partnership [5].

Indirectly, supporting physicians, clinical trial recruitment, and biospecimen donation research can benefit underserved communities, but this further detail dissipates the direct focus on underserved communities. As an aside, the Outreach Core is identified under Research Strategy, but provides the following advisement:

*Note:* Hypothesis‐driven outreach research projects are encouraged and allowed for this FOA; they must be described under Full Research Project or Pilot Research Project components, as appropriate, NOT in this Outreach Core component [5].

Providing one more example to demonstrate the ambiguous guidance for community‐oriented cores within NIH, the National Institute of Minority Health and Health Disparities' (NIMHD) Research Center for Minority Institutes (RCMI) program announcement similarly indicates that the required Community Engagement Core should support research projects in recruitment and retention of study participants with community organizations, develop and implement dissemination strategies for a broad range of audiences, and translate findings into sustainable community and system‐level changes at the local level and beyond. This directive suggests that the Core should equally weigh its efforts on community partnership building, dissemination, participant recruitment, and translational research. The expertise needed for participant recruitment and retention is quite distinct from translation science. Ramandadhan et al. [6] calls for more dissemination and implementation science in Cancer Centers, indicating the range of tasks is too expansion for Outreach.

Considering the mixed directives for developing NIH‐supported community‐facing cores, successfully funded community outreach cores often use a variety of strategies to benefit and engage the community and to disseminate and translate research outcomes. This diversity supports innovation and an opportunity to tailor approaches to local and regional communities. However, the range of strategies often makes it difficult for NIH‐funded community outreach cores to collaborate. The weight of their emphasis‐approved core components, that is, community education, research participant recruitment and retention, research dissemination, and translational science, can yield quite different aims and objectives.

The diversity of outreach core activities is evident in a brief overview of published activities and outcomes, which include a broad array of educational and research activities with a significant breadth of stakeholders. A handful of manuscripts report educational activities [6, 7, 8], while others report the outcomes of pilot research related to community readiness and attitudes toward cancer [8, 9] and dissemination of evidence‐based behavioral interventions [8, 10]. Reports may focus on underserved communities of interest, while also centering activities on clinicians, researchers, and/or trainees [8]. Community outreach and engagement cores at NCI‐designated cancer centers have identified challenges in collaborating across centers and the collection of metrics [11], while the C‐PACHE mechanism has facilitated some collaboration across centers to identify outreach measures [12].

## 4 Methods

### 4.1 Strategies

Over time and with changing leadership, the NACP Outreach Core has employed many strategies. Most have centered on engaging Arizona Native Nations in discussions about cancer‐related needs, identification of tribal liaisons as NACP points of contact, community education, capacity building to enhance knowledge of cancer risks and the value of screening, community readiness for cancer programming and research [9], and support for families navigating treatment regimens. The strategies shared here were implemented in the 2019–2024 funding cycle. The Outreach Core during this cycle consisted of two faculties (one native American, one non‐native), two Master of Public Health (MPH)‐level native American program coordinators, five native American graduate students and one native American undergraduate student.

During the 2019–2024 funding cycle, NACP Outreach Core had the following goals: (1) Increase the institutions' researchers' and students' knowledge of native Americans' cancer burden and programming to enhance capacity to design sustainable, relevant cancer research and prevention programs; (2) train NACP‐supported students and investigators in best research practices when engaging in research with tribal communities; (3) facilitate community‐based dissemination of NACP research results by training students and investigators; and (4) collaborate with NACP, institutional, and community partners to develop, prioritize, and implement requested cancer‐related activities to benefit native American communities.

**Goal 1**: Increase researcher, trainee and tribal communities’ knowledge of native American cancer‐related issues to enhance institutional and tribal capacity to design sustainable, relevant cancer research and prevention programs

### 4.2 Indigenous Cancer Prevention Speaker Series

The first of three approaches under Goal 1 was the Indigenous Cancer Prevention (ICP) Speaker Series developed to disseminate the accomplishments of tribal‐based cancer programs and current research or innovative programs addressing native American cancer health equity. The series offered 2 presentations each academic semester (Fall and Spring). The series was designed to be accessible, informative, and engaging to a broad audience. The attendees included students of all levels, university faculty and staff, community members, cancer survivors, service providers, and tribal and community lay health workers.

### 4.3 Summer Internship for INdigenous Peoples in Genomics

The second approach under Goal 1 was collaborating with the Summer Internship for INdigenous Peoples in Genomics (SING) program. The SING program includes an annual one‐week short course with hands‐on training in molecular biology, bioinformatics, bioethics, and the ethical, legal and social issues that often arise in genetic studies with Indigenous peoples. The goal of the SING program is to foster a new generation of intellectual leaders who will define the expanding frontiers of genomic analysis with a specific focus on research within Indigenous communities [13]. NACP leveraged resources and partnered with Diné College (Navajo Nation Tribal College) to host SING at NAU (in 2023).

### 4.4 Education

The third approach was the design and dissemination of printed and online 4‐page pamphlets (referred to as volumes) as resources to help guide researchers and tribal communities to learn and understand research practices, cancer terminology, and current cancer screening recommendations. Four pamphlets were created in cycle 3, and 6 were created in cycle 4 (the current cycle). All 10 volumes were distributed to tribal leadership and health directors across the state in the current funding cycle. Volumes I–III provide guidelines on understanding and protecting Tribal Sovereignty and Building Tribal Partnerships; Volume IV offers Guidelines for Researchers planning to engage with tribal communities; Volumes V–X focus on Native American Specific Cancer Incidence and Mortality Rates, caregiver tips, clinical trials, cancer survivorship, and childhood cancer. Volumes I–III materials were created in cycle three of the funding cycle in partnership with the Hopi Tribe Native American Research Center for Health Partnership grant (NARCH S06GM12801).

**Goal 2**: Training in research best‐practices when collaborating with tribal communities

### 4.5 Native American Research Protections and Biospecimens Protections Workshops

In 2018, the Arizona Board of Regents (ABOR) implemented a Tribal Consultation Policy (policy 1–118) outlining the expectations and requirements of Arizona university employees when engaging Native Nations, by recognizing fundamental principles of tribal sovereignty, consultation, and respect [14]. Partnering with Native Nations for capacity support, research and outreach, is unique in the U.S. due in large part to the legacy of historical trauma, harmful research practices and tribal sovereignty [14]. As Sovereign Nations, tribal leadership has the right to establish laws governing the behaviors of their citizens, tribal lands (reservation) residents and non‐citizens working on reservation lands [14]. For researchers and external partners, sovereign jurisdiction and authority extend to approval or rejection of all components of research, and generally require regular progress reports, establishment of signed data ownership agreements, and prior authorization of public dissemination activities (e.g., presentations and manuscript submissions) of research outcomes; these parameters apply to de‐identified and aggregate data.

The NACP Outreach Core workshops offered institutional and national training to educate researchers about Native Nation sovereignty, data ownership, especially in regard to biospecimens, and respectful engagement and collaborative dissemination with native American communities. The approach included ongoing workshops in Native American Research Protections and Considerations as part of the Responsible Conduct of Research Program and a workshop on Considerations in Biospecimens Protections when working with Tribal Communities. These workshops were part of a larger series at the University of Arizona and reached faculty and students across campus. UACC NACP Outreach Co‐lead co‐taught these sessions with the UA Assistant Vice President for Tribal Relations, and the Director, UA Research, Discovery and Innovation, Native Peoples Technical Assistance Office.

**Goal 3**: Facilitate community‐based dissemination of NACP research results

### 4.6 Community Dissemination and Application Training (C‐DAT)

To improve institutional and investigator capacity to conduct relevant and respectful research with Native Nations, NACP Outreach developed the C‐DAT. C‐DAT is a technical assistance program designed to augment NACP research teams' skills in disseminating knowledge and findings to communities and community programs, focusing on native American communities. Dissemination and application of new knowledge into practice proceeds through three stages, from awareness through acceptance to adoption [15]. To make dissemination and application effective, developers [16, 17, 18] of training programs recommend including: (1) structured case‐level feedback; (2) practice in context early in the research phase, ideally with community partners; and (3) deliberative practice when new knowledge is formed. Concurrently, the Evaluation Committee of the Association for Clinical Research Training has stated that training must be flexible enough to accommodate the needs of individual institutions and trainees yet rigorous enough to document meeting short‐, intermediate‐, and long‐term objectives and pre‐established competency requirements [19]. Applying this stage training approach and focusing on dissemination components most relevant to NACP research projects, specifically communication, cultural relevance, and community engagement, the Outreach Core developed C‐DAT grounded in the three stages of transferring knowledge to application, awareness, acceptance, and adoption [15] (Table 1). The goal is to build dissemination and application research skills in NACP students and investigators to provide experience with research's potential to enhance healthcare services, community‐relevant cancer education, and community‐engaged cancer research.

**TABLE 1:** Community dissemination and training (C‐DAT) framework.

Stage	Approach	Activities	Competencies
Awareness (short‐term)	Didactic Interactive workshop	Overview Guided team activities to identify audience characteristics and research outcomes to disseminate. Case study reviews, and critique of existing materials designed for native American audiences	Communicate research findings to native American audiences, lay public, and media Propose culturally relevance to messages and graphics Translate implications of research to clinical practice, community leaders, health guidelines, and funding agencies Draft policy briefs for consideration by community partners
Acceptance (mid‐term)	Practice	Technical assistance: investigators, community partners, and outreach team develop communication/implications strategies and draft policy briefs	
Adoption (long‐term)	Deliberative Practice	Implementation of two more dissemination/translation strategies	

Stage 1 of C‐DAT is an interactive 3‐h workshop based on the W.F. Kellogg Foundation's Elements of a Strategic Communication Plan [20]. This plan has six steps: (1) determine goal, (2) identify and profile audience, (3) develop messages, (4) select communication channels, (5) choose activities and materials, and (6) establish partnerships for dissemination. C‐DAT presents the steps: after introducing each topic, the research team has 10 min to develop a project‐ specific response to specific step. Teams were then invited to share their responses to the other partnering research teams. Although the sharing activity added to the length of the training, hearing other teams' ideas expanded investigators' realm of dissemination strategies.

The NACP Outreach team modified the Kellogg Foundation's document by adding information on making messages culturally relevant, particular to native American audiences in the U.S. Southwest. For example, some health messages state that adhering to cancer screening recommendations should be part of a self‐care regimen. In many native American communities, family is a priority and may take precedence over individual needs. NACP C‐DAT suggested modifying health messaging to highlight prevention in the context of family, for example, screening to ensure that “you stay healthy so you can take care of your family and loved ones.” Similarly, C‐DAT encouraged research teams to initiate Step 6 before agreeing on their dissemination plan. For teams that had community partners integrated into their teams, those partners also attended C‐DAT. Teams that did not have community partners were encouraged to meet with the NACP Outreach team to gain additional guidance for their plans.

NACP added an appendix to the modified Kellogg Foundation training to provide examples of graphic materials developed specifically for native American audiences. Some examples adhered to the principles of tailoring messages to an audience and cultural relevance, and others did not. At the end of the workshop, the Outreach team facilitated a session inviting workshop participants to critique the materials using the new skills they had gained in completing Stage 1. After the workshop, teams were tasked with confirming their plan, developing a timeline for implementation, and meeting with 1–2 Outreach Core members to discuss their plans (Acceptance) and outline the logistical steps for action (Adoption).

**Goal 4**: Collaborate with communities in the development and implementation of cancer‐related information activities to benefit native American communities

### 4.7 Community Engagement to Understand and Support Educational Needs

As mentioned previously, the faculty and staff of the Outreach Core had professional relationships with employees of tribal health departments prior to NACP and/or in the contexts of other non‐NACP partnerships. These connections were often reinforced by NACP staff and faculty accepting invitations to participate in community break‐out sessions or offering to set up a resource table to provide educational materials about cancer screening and behavioral risk factors. Promotional items or swag such as canvas bags, lip balm, sunscreen and pens imprinted with the NACP logo and website, were available at the table to attract visitors and to provide contact information. NACP's willingness to engage in community events was offered in the spirit of reciprocity even with communities that did not have formal relationships with NACP, demonstrating a sincere commitment to cancer health equity in native American communities.

NACP presence in the community supports informal conversations with professional and lay health educators about the need for native American specific cancer information and popular information formats. For example the value of podcasts featuring native American hosts and speakers and developing more engaging website materials was discussed. In 2020, with the COVID‐19 induced travel and gathering restrictions, NACP Outreach Core staff and faculty reached out to a CDC funded tribal breast and cervical cancer prevention program to propose and develop the first NACP podcast. The effort was designed to continue virtual collaborative activities and community education [21].

### 4.8 Continued Engagement With the Community Advisory Board

The 6 to 8‐person CAB, representing several Arizona Native Nations, serves the entire NACP partnership. The Outreach Core works with the CAB to inform specific activities. One such example was the development of a review process/feedback matrix for the CAB to assess cancer research projects submitted in response to NACP's call for proposals. The CAB review occurs within the larger scientific review process which engages external investigators who use the NIH scoring/review criteria [22]. CAB review process included strengths and weaknesses for each of the following categories: (1) does this project advance our knowledge, understanding and/or prevention of cancer within Tribal communities?; (2) is the team proposing to complete the work have experience and/or use a culturally appropriate approach to work with Tribal communities?; (3) is the investigative team proposing new ideas or approaches that benefit Tribal communities?; (4) do the steps the research team is proposing make sense and propose maximum benefit to Tribes and Tribal communities?; (5) does the research team describe resources that will ensure it accomplishes the goals of its project?

In addition, Outreach Core members attend the CAB meetings on a regular basis to update the CAB on Outreach activities and to gain input on specific strategies (e.g., C‐DAT) and to provide an overview of NACP funded activities.

### 4.9 Evaluation Approaches

Evaluation plans aligned with each of the respective Outreach Core activities. Two approaches were organized, facilitated and evaluated by external entities so the findings are excluded from this paper, specifically the Summer Internship for INdigenous Peoples in Genomics/SING program and the workshop series as part of the UA Responsible Conduct of Research Program, the *Tribal Consultation and Engagement with Native Communities* (noted by an asterisk in Table 2).

**TABLE 2:** Outreach approaches and evaluation domains (2019–2024).

Outreach approaches	Evaluation domains
Indigenous Cancer Prevention (ICP) webinar series	Attendee position and if attendees work for a Tribal serving institution Organizational reach/U.S. states reached Coded types of organizations engaged (nationally)
Summer Internship for INdigenous Peoples in Genomics (SING) faculty speakers^a^	Partner with tribal college to co‐host SING at NAU These events were externally organized/managed/evaluated, no evaluation findings are provided in this paper.
Dissemination strategies: Education materials and podcasts	Number of podcasts/topics, listener reach (geographic), and modality Cancer education pamphlets (community dissemination/reach)
Native American Research Protections and Biospecimens Protections workshops^a^	Track workshop topics and attendance numbers Pre/post knowledge change (These events were externally organized/managed, no evaluation findings in this paper)
Community Dissemination Application Training (C‐DAT)	Level of experience with dissemination (community) Agreement statements/Likert scale (subject matter, materials, facilitation, did content match skill level, engagement activities) Agreement statements/Likert scale (learned new information, better prepared to convey outcomes to community/apply research findings to improve community health) based on C‐DAT competencies. Agreement statements/Likert scale (usefulness of the materials) Changes in practice resulting from C‐DAT (qualitative)
Community Engagement Activities	Number/type of organizational collaborators Audience, event type, and cancer focus External competitive grant funding secured CAB research proposal review (community focused review questions) CAB feedback on the NACP research proposal review process (agreement statements, Likert scale)

^a^ The evaluation results for these activities are not included in this paper. Another entity conducted those evaluations.

## 5 Results

All products discussed below can be found on the NACP websites [23, 24].

**Goal 1**: Increase researchers' and students' knowledge of native Americans' cancer‐related issues to enhance institutional and tribal capacity to design sustainable, relevant cancer research and prevention programs

### 5.1 Indigenous Cancer Prevention Speaker Series

A total of 20 speakers, 17 (85%) of whom represented a tribal nation, presented through a Zoom^tm^ webinar platform. The speaker series was intended to be in‐person format, but due to the restrictions of the COVID‐19 pandemic, all sessions were presented online. Speaker organizations represented an array of organizations/entities including Native American Serving Organization (non‐profit); American Cancer Society; CDC funded Breast and Cervical Program; Center for Disease Control; Comprehensive Cancer Center; Equity focused Cancer Organization; Federally Qualified Health Center; Higher Education institution; Regional Tribal Health Board; Tribal Cancer Center; Tribal Nation Research Office; and a Tribal Tobacco Cessation/Prevention Education Program. Table 3 provides an overview of the ICP series including date, title, number of attendees, and a brief overview of each webinar.

**TABLE 3:** Indigenous cancer prevention speaker series.

Date	Title	Attendees	Overview
*Year 1*			
11/25/19	Cancer Trends in Indian Country	23	Native American Surgical Oncologist presented cancer data for native Americans with a special focus on liver cancer
02/12/20	Cancer among Alaska Natives	18	Focus on Alaska Native cancer data, prevention, and research, Alaska Native Tumor Registry
03/11/20	Quality of Life among American Indian Cancer Survivors	34	AIAN cancer data in the Southwest, cancer survivorship, and quality of life
04/09/20	Human papillomavirus (HPV) and Cervical Cancer	63	NAU student presented and outlined the findings of her project relating to HPV and Cervical cancer
*Year 2*			
10/07/20	American Indian Cancer Foundation (AICAF) Program	18	Information on cancer burden, AICAF cancer plan and resources and cancer screening recommendations during the COVID pandemic
11/04/20	Data to Cancer Prevention Activities	33	Overview of Urban Indian Health Institute (UIHI), American Indian/Alaska Native (AI/AN)^a^ cancer data, rates, and findings, interventions and cancer plans; partnership with AICAF
02/25/21	Indian Health Services, and the Cancer Care Continuum	47	Understanding the meaning of relationships with Indian Health Services, Rural Safety Net Providers, and the cancer care continuum
04/30/21	Understanding Pediatric Cancer—Personal Experience	14	A NACP Outreach Core member talked about her pediatric cancer journey from a parent's point of view
*Year 3*			
11/10/21	Select Cancers in Indian Country and Cherokee Nation	18	The director of the Cherokee Nation Health Research provided cancer data based on the Cherokee Nation Cancer Registry
11/17/21	Prevention So That People May Live	16	The Great Plains Tribal Leaders Health Board members outlined their organization and their cancer‐related programs
02/18/22	Women's Journey toward Cancer Prevention	25	AIAN cancer screening and prevention – Breast, Cervical, and Colon cancer presented by HOPI Cancer Support Services
03/09/22	Radiation Exposure Compensation Act	17	Health Promotions Program Manager with North Country HealthCare and an NACP CAB member explained the process and guidelines of the Radiation Exposure Screening and Education Program
*Year 4*			
10/24/22	Salish Cancer Center—Resource Sharing	41	Establishment of the integrative cancer center with the Puyallup tribe for tribal and non‐tribal patients.
11/08/22	Navajo Nation—Air is Life Act of 2021	13	The program manager for the Southwest Navajo Tobacco Education Prevention Project explained of the coalition and policy, tobacco and health, and passing of the act
02/27/23	Through the Lens of the American Cancer Society (ACS)	23	AIAN^a^ cancer burden with a focus on colorectal cancer; ACS advocacy, research, and patient support
04/05/23	Cancer Lifeline	27	The executive director of Cancer Lifeline provided information relating to cancer support group and cancer patient and caregiver/family resources
*Year 5*			
09/25/23	Native American Cancer Surveillance and Mortality	19	An epidemiologist in the Division of Cancer Prevention and Control at CDC led a talk about IHS linkages, AIAN cancer incidence database, and cancer among the Navajo
10/30/23	Northwest Tribal Comprehensive Cancer Program	26	Background information of various Northwest Portland Area Indian Health Board cancer programs and activities; Northwest specific cancer data
03/26/24	Addressing Psychosocial Oncology Concerns	31	Oncology social worker described psychosocial services to help improve the wellbeing of cancer patients and their family/caregivers
04/26/24	Cancer in Native American Children	28	Tribal health center nurse practitioner discussed diagnosis risk and survival, disparities, and long‐term quality of life of AIAN children with cancer

^a^ Federal data reports use the acronym AI/AN; subsequently in this manuscript AI/AN will only be used when citing federal sources.

From 11/25/19 to 04/26/24, the series reached 824 attendees (534 in real time on the session day; 290 viewed the session recordings via YouTube). Attendees participated from 33 U.S. states as well as Canada. ICP webinars spanned multiple groups including 112 Tribal community health workers/CHRs (non‐CHR *n* = 56); CHR *n* = 56; 99 Faculty (all levels); 85 Students (Undergraduate *n* = 43; Graduate *n* = 42); 76 attendees reported “other”; 49 service providers; 36 community members; 31 university staff; 9 Postdoctoral Scholars; 9 cancer survivors; and 28 attendees did not respond to this question (missing data). Attendance counts are not unique; that is individuals could have attended multiple events.

The series reached a total of 106 distinct organizations including: native American serving organizations (*n* = 27), tribal entities (*n* = 26), higher education institutions (*n* = 26), and health organizations (non‐tribal [*n* = 14]; federal/state departments or organizations [*n* = 8]; and other organizations [*n* = 5]). Several key examples include Tribal Epidemiology Centers, Federally Qualified Community Health Centers, and Urban Indian Organization/Health Programs. About 50% of attendees reported working with native American serving and tribal‐serving entities. The speaker series recordings are available for viewing on the University of Arizona NACP website [8].

**FIGURE 2:**
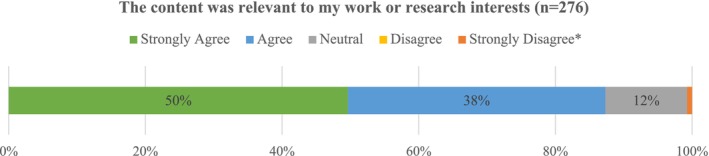
ICP webinar evaluation: content was relevant to my work or research interests. *1% of respondents strongly disagreed.

Attendees completed an online pre‐ (at the time of registration) and post‐ (after the session) evaluation to measure knowledge change. The presenters were required to prepare knowledge‐focused questions to align with their presentation content; thus, each of the 20 ICP sessions had different pre/post questions. The Outreach team led the effort to assess knowledge change among attendees and managed the pre/post assessments and prepared corresponding reports. Given the breadth of attendees (nine different types ranging from cancer survivors to students) and the fact that each of the pre/post knowledge assessments was different, the team chose not to present the knowledge change findings by participant type. Collectively (across all 20 ICP webinars) attendee knowledge increased by an average of 8.2%. The level of knowledge change varied across ICP webinars. In some cases, there was a significant increase (over 35% change); while in other cases there was a negative change. Given that each respective speaker (content expert) was asked to create their own set of content‐based knowledge questions, a limitation of this approach was controlling the quality of the questions and ensuring the questions aligned with the content covered. An evaluation question that provided insight on the value of the series was attendees' level of agreement with the statement: *The content was relevant to my work or research interests*. Figure 2 illustrates the cumulative responses from 18 out of the 20 surveys (two early surveys did not include these questions). Nearly 90% of respondents either *strongly agreed* (50%) or *agreed* (38%) with the evaluation statement.

**FIGURE 3:**
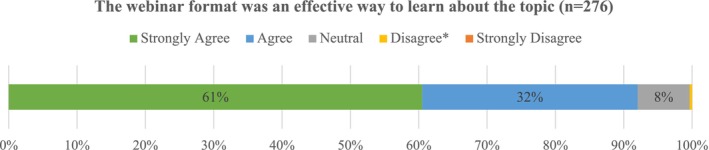
ICP webinar evaluation: the webinar format was an effective way to learn about the topic. *0.36% of respondents disagreed.

Attendees reported their level of agreement with another statement: The webinar format was an effective way to learn about the topic. Figure 3 includes cumulative findings across 19 out of the 20 surveys (one survey did not include this question). Over 90% of respondents either strongly agreed (61%) or agreed (32%) with the evaluation statement.

### 5.2 Summer Internship for INdigenous Peoples in Genomics

The goal for this activity was for NACP to leverage resources and partner with a local tribal college to host SING at NAU. In the summer of 2023 NACP and Dine College (Navajo Nation Tribal College) partnered to host SING at NAU. The SING program is funded through a separate grant at another institution, so the goal here is not to share program results but to demonstrate a successful collaboration with a local tribal college. SING invited NACP to participate in a series of professional development opportunities supporting Indigenous leadership and advancing Indigenous community collaborations in genomic research.

### 5.3 Community Dissemination (Education Materials)

In the 2019–2024 grant period, the community education materials (Volumes I–X) were disseminated to all 22 Arizona tribal leaders and tribal health directors as well as the First Lady of the United States, Dr. Jill Biden; the Secretary of the U.S. Department of Health and Human Services, Xavier Becerra; and at select outreach events designed for predominantly native American audiences. The correspondence with the tribal leaders included a pre‐stamped feedback postcard and a QR code regarding the materials' usefulness. Two tribal health organizations responded and provided feedback from the postcards. Another tribal organization called to express its appreciation of the materials. The education materials are available for viewing on the University of Arizona NACP website [23].

**Goal 2**: Enhance skills and competency of NACP‐supported students and investigators regarding best research practices when engaging in research with tribal communities

### 5.4 Native American Research Protections and Biospecimens Protections Workshops

The workshop series was organized and hosted by the UA Responsible Conduct of Research Program. The pre/post knowledge evaluation was conducted by UA (not NACP). As such the findings are not part of the results being disseminated in this paper. The training includes diversity with respect to native American communities, UA Native Peoples Technical Assistance Office, Institutional Review Board, Contracts, and Research with Biospecimens. Collectively, 16 workshops reached *n* = 517 people.

**Goal 3**: Facilitate community‐based dissemination of NACP research results

### 5.5 Community Dissemination and Application Training

In the summer of 2020, the Outreach team delivered the first stage of C‐DAT virtually to one research team via Zoom^tm^. This delivery method was necessitated by COVID‐19 restrictions to avoid gathering. Since its launch, 31 investigators (including nine funded pilot/full project teams 2019–present) have participated in Stage 1 of the C‐DAT, an interactive workshop introducing messaging strategies and community dissemination venues. Stage 1 included a workshop evaluation feedback to enhance the program. 84% (26/31) respondents who completed the evaluation reported, “I feel better prepared to work with communities to apply my research outcomes to inform practice and or policy to improve community health.” Workshop evaluations included questions about materials/content and approaches. There was a high level of agreement (over 90%) for the majority of metrics (Figure 2). One metric indicated slightly lower agreement (77%) noting the timing of the workshop needed to be adjusted to align with research project timelines. The Outreach team shifted the timing as a result.

All four NACP pilot projects that are required to participate advanced to Stage 2: Acceptance (planning). In the fall of 2022, the outreach team facilitated technical assistance sessions with each of the four active NACP research project teams. During these sessions, the evaluation team documented support that projects requested from outreach. Resulting dissemination support outreach provided to projects includes review of recruitment materials, review of informed consent documents (reading level), guidance on when/how research projects can engage with the CAB, share best practices for dissemination to tribal communities, and identify a Native student that can support projects at the community partner site. During the piloting stage of the C‐DAT, one NACP full project completed all three stages by deliberately producing and disseminating a well‐woman video that reached over 584 people.1 In the successive funding cycle (2024–2029), NACP required all funded projects to complete all three stages of the C‐DAT.

Based on evaluations and verbal feedback, research teams expressed frustration with limited financial resources to launch community dissemination efforts and limited knowledge of specific, community‐based dissemination venues and contact information. To ensure NACP continued its commitment to serve community partners and to address the need for research teams' financial resources, NACP's forthcoming call for proposals for new research projects will require that researchers acknowledge their willingness to complete C‐DAT and budget a minimum of $1000 to support community dissemination activities (e.g., travel to community meetings, publish a story in a tribal newspaper or develop a story for a tribal radio station). In 2023, NACP realized many researchers new to working in tribal communities were not familiar with local and tribally controlled resources for reaching native American audiences. To address this gap in information, the NACP Outreach Core developed a Directory for Reaching Relatives Using Media (DRRUM) as a resource to support research teams during Stages 2–3 of C‐DAT. DRRUM is a comprehensive, online, dynamic list of all Arizona tribally administered and non‐tribal but regionally local radio stations, newspapers, and newsletters, including the cost of using the resources and contact information, for example, phone numbers and emails. This resource is new, and the value to the investigative team has not yet been assessed.

**Goal 4**: Collaborate with communities in the development and implementation of cancer‐related activities to benefit native American communities

### 5.6 Community Engagement

Over the course of the 5‐year funding cycle, the Outreach Core maintained and expanded relationships with state and tribal cancer programs and secured external funding to advance native American cancer health equity in Arizona. An accomplishment that underscores the strength of the relationships formed through the NACP is a successful 4‐million‐dollar American Cancer Society award to NAU, led by the NAU Outreach Core, the Center for Native American Cancer Health Equity (C‐NACHE). C‐NACHE is distinct from NACP in that the recommendations and voice of the participating Arizona Native Nations represented on the Community Advisory requested the research project (i.e., not investigator initiated) and developed strategies to propose state and national policy changes that could improve cancer care through the continuum for native Americans. C‐NACHE includes seven native American individuals affiliated with the NACP team. Notably, the three research projects funded with the C‐NACHE are directly related to NACP funded research and/or relationships cultivated through NACP: Project 1: Environmental Exposures from Legacy Mining as a Social Determinant of Health Linked to Kidney Cancer; Project 2: The Navajo Cancer Workgroup: Enhancing impact of epidemiological data for cancer prevention and control among the Navajo people; and Project 3: My Health, My Choice: Adapting and Testing a Cancer Screening Education program for Native women with Intellectual and Developmental Disabilities [25].

Despite a global pandemic, Outreach collaborated with 9 local organizations: Arizona Department of Health Services (AZDHS), Community Outreach and Patient Empowerment (COPE), Hopi Cancer Support Services (HCCS), Native Americans for Community Action (NACA), Navajo Nation Human Research Review Board (NNHRRB), NN Breast and Cervical Cancer Prevention Program (NNBCCPP), North Country Healthcare (NCHC), Tuba City Regional Health Care Corporation (TCRHCC), and UA Native American Advancement & Tribal Engagement. Collectively Outreach conducted 20 community cancer outreach events including cancer town hall meetings, health fairs, radio segments, and cancer‐related fun runs (awareness), reaching over 1400 community members. Notably, Outreach collaborated with NACA and the NNBCCP to organize a mobile mammography screening. Most of these events were collaborative, where NACP worked with the local organizations listed above to support their community events. In collaboration with an Indigenous Oncologist at UACC, Outreach created two YouTube videos focused on the importance of men's and women's wellness exams for cancer prevention. These educational videos have reached over 750 views to date. Collaborations with UACC Community Outreach and Engagement (COE) included several fun run events reaching urban Indian community members; a cancer conference, Beyond Cancer Navigating Care, geared toward cancer survivors, caregivers/co‐survivors, family, friends, and community members; and a town hall in Flagstaff, Arizona focused on cancer policy needs.

The Outreach team collaborated with leadership to develop a community review matrix for research projects being proposed within the NACP. An evaluation was conducted with CAB members (*n* = 9) who participated in the research review process. CAB members responded to a series of questions about the review process using a Likert scale to indicate their level of agreement (Strongly agree, agree, disagree, strongly disagree and not applicable). All CAB members reported feeling comfortable reviewing the proposal they were assigned (56% Strongly agreed and 44% agreed). CAB members were asked to share their advice for how the NACP could improve the CAB proposal review process. Responses were qualitatively coded and summarized. Applicants should provide an overview figure or lay language to summarize the projects and should better specify how community engagement will be done. CAB requested more time to review the proposals (before the meeting) and additional time during the review session to discuss each proposal.

The first completed podcast was posted on Buzzsprout^tm^ and Spotify^tm^ and the NACP website [26]; within 2 months, the podcast was downloaded more than 50 times by listeners in the US Southwest. Given this reach, NACP Outreach Core was motivated to develop a series to provide cancer‐related education and personal stories specifically by native American public health and health practitioners, and for native American audiences. In 2021, NACP Outreach established *The Taking Care of Us* series. By May 2025, seven episodes were available (253 downloads) [24]. The podcast series has been promoted on two tribal radio stations and is available on Spotify^tm^ and YouTube^tm^. The *Taking Care of Us* series has reached listeners in 21 U.S. states and 65 cities in North America and Europe. Internationally, listeners in the Netherlands, the Faroe Islands, Australia, and New Zealand have downloaded one or more of the podcasts.

As mentioned, the first episode featured the staff of a tribal breast and cervical cancer program, who described their screening and support services. Four episodes disseminate the outcomes of NACP‐funded research projects. Other episodes include an interview with a native American mother who describes navigating health care systems, family emotions and strategies to get a cancer diagnosis and treatment for her child, and one episode is interviews with activists and advocates for Missing and Murdered Indigenous Relatives (MMIR). Although the latter episode is not cancer‐specific, the reauthorization of the Violence Against Women's Act in March 2022 was lauded by tribal leaders as landmark progress for the MMIR movement and aligned with the podcast series' overarching objective of featuring stories of self‐determination related to health [27]. Interviews, editing, and production were done by NAU MPH graduate students, a majority of whom are native American and supported by NACP.

## 6 Discussion

The broad language in the FOA, which centered on “outreach efforts that would benefit communities with cancer health disparities” provides necessary flexibility to respond to communities' identified needs and interests. At the same time, differing foci across outreach teams makes it challenging to measure impact in a way that is meaningful to the community of science. Data collection should be acceptable and feasible within a particular community context, and at times, the collaboration necessary for engaged work may limit the cores' ability to collect data. These limitations necessitate creativity and sensitivity to communities' history with research. While not in the FOA language, Outreach Cores are often responsible for establishing and maintaining community trust that enables research collaboration. Yet, an expectation that Outreach Cores should be tools to keep the research enterprise moving is not feasible, as their relationship focuses on open communication and reciprocity rather than extraction.

The Outreach Core's focus on information sharing meets an identified need, but metrics are difficult to identify in the extant literature. Descriptions of cancer education efforts or interventions to enhance awareness of cancer risks or screening recommendations to native American audiences are limited. In a systematic review of interventions to reduce disparities in cancer screening, only one report specifically discussed working with native Americans [28] Innovative approaches to cancer information sharing with native American communities include the use of text message reminders to complete colorectal cancer screening [29] and a cancer‐focused webinar series that reached more than 22,000 viewers during the COVID‐19 pandemic, with 17% identifying as native American [30]. The Centers for Disease Control and Prevention [31], Indian Health Service [32], the American Indian Cancer Foundation [33], Roswell Park Comprehensive Cancer Center [34] and Native American Cancer Initiatives Inc. [35] have websites providing cancer information and in some cases printable educational material for native American populations but the frequency with which these sites are used (“hits”) or the downloads of available materials is not reported in the literature.

NACP Outreach has piloted a range of strategies to provide cancer related information to tribal community members, lay health educators, providers and researchers. Several strategies were highly successful in meeting or exceeding the specific goals of the NACP Outreach Core. An unintended (positive) outcome of shifting the ICP webinar series from in‐person to an online format (due to the COVID‐19 pandemic) was expanding the reach nationally to 106 organizations across 33 U.S. states. If the webinar had been in‐person, reach would have been restricted to local participants in southern Arizona. The C‐DAT technical assistance phase supported strong integration of research projects with Outreach Core meeting the NCI expectation for integrated programming. Furthermore, the C‐DAT deliberative practice phase resulted in collaborative work to enhance research project engagement with community dissemination efforts such as the identified need for the DRRUM and education videos that align with NACP research foci. One of the important innovations of the Outreach team during the 2019–2024 funding cycle was the development/implementation of a modified review matrix for research projects to provide the CAB an opportunity to review and leverage their expertise to inform the development of relevant and culturally appropriate research projects. One of the major successes of the Outreach team is leveraging NACP relationships (social capital) to obtain an American Cancer Society award (Center for Native American Cancer Health Equity) is in direct alignment with the NCI goal for CPACHE programs (like NACP) to achieve sustainability by obtaining competitive external support. The Outreach team's regular connection with native American communities described briefly without the context of the CAB, is an invaluable source of inspiration to try new approaches to educate communities, providers and researchers.

In reflection, the Outreach and Evaluation teams identified several opportunities to improve future outreach and evaluation approaches. Given the somewhat low rate of knowledge change (8% average increase) for the ICP webinar and the challenges with quality control (whereas each speaker provided pre/post questions based on the content they presented), future evaluation questions would be centered on evaluation questions calibrated with the intended goals or learning objectives of the webinar series (collectively) rather than individual webinar content. The technical assistance component of the C‐DAT was well received. However, organizing single events where all research team members could attend and syncing those events with multiple research projects' readiness to initiate dissemination efforts, was challenging. As a result in the new funding cycle (2024–2029), the Outreach team is shifting from a one‐time event (engaging all research projects) to a standing (quarterly) community dissemination strategy meeting where teams can request specific guidance or technical assistance to conceptualize or implement dissemination strategies.

One limitation of the work presented in this paper was the lack of impact‐focused evaluation related to community engagement with local organizations. The evaluation team is planning an impact evaluation (using ripple effect mapping) to document the term impacts of NACP's work with long‐term community partners such as HCCS, NACA, NNBCCPP, and NCHC. Due to the challenges of collecting data to better understand cancer‐related behavior change affected by outreach activities, Outreach might develop collaborative activities with communities to measure impact, such as “social marketing” projects to measure messaging impact [10]. Further, institutional metrics might offer insight into the impact of researcher‐focused trainings, such as measuring an increase in the number of researchers working with tribal communities.

## 7 Conclusion

The outreach activities of NIH‐funded centers vary, making the collective impact of Outreach programs difficult to assess. The ability to evaluate outreach approaches, document success, and demonstrate outcomes and impacts should be considered when designing outreach strategies to document outcomes and communicate impact. From 2019 to 2024, the NACP Outreach Core has implemented cancer education strategies designed to reach native American audiences, primarily in Arizona and structured guidance for NACP‐funded research teams working on cancer‐related concerns disproportionately impacting native American populations. Through its efforts, the NACP Outreach Core's foci has evolved to develop accessible resources for native American communities, health educators, providers and even researchers. Moving into the next funding cycle (2024–2029) the NACP Outreach team will employ strategies that will strengthen Native Nations' capacity to advance cancer health equity while documenting more robust outcomes and impacts.

## Author Contributions

**Nicolette I. Teufel‐Shone, Carol Goldtooth, Francine C. Gachupin,** and **Jacquanette R. Slowtalker:** conceptualization of manuscript. **Kelly A. Laurila, Carol Goldtooth,** and **Jacquanette R. Slowtalker:** methodology and data analysis. **Kelly A. Laurila, Janet R. Yellowhair, Ashley D. Lazaro, Eli BigThumb,** and **Alexis K. Talayumptewa:** data acquisition. **Nicolette I. Teufel‐Shone, Carol Goldtooth, Francine C. Gachupin, Jacquanette R. Slowtalker,** and **Kelly A. Laurila:** writing – original draft preparation. **Nicolette I. Teufel‐Shone, Carol Goldtooth, Jacquanette R. Slowtalker, Kelly A. Laurila, Alexis K. Talayumptewa,** and **Julie S. Armin:** writing – review and editing. **Nicolette I. Teufel‐Shone** and **Francine C. Gachupin:** project supervision. **Nicolette I. Teufel‐Shone** and **Francine C. Gachupin:** funding acquisition. All authors have read and agreed to the published version of the manuscript.

## Ethics Statement

Ethical approval was obtained from Northern Arizona University (IRBNet ID # 1535642). Evaluation research activities are approved by the NAU Institutional Review Board. Non‐generalizable outreach evaluation activities, not considered research, were listed in the approved IRB protocol as being excluded from the evaluation research.

## Consent

The authors have nothing to report.

## Conflicts of Interest

The authors declare no conflicts of interest.

## Data Availability

Data supporting this study cannot be made available as it is part of the evaluation records, not intended for generalization/research.
